# Inflammatory, angiogenic and neurogenic serum biomarkers in patients with patellofemoral pain compared to healthy controls

**DOI:** 10.1016/j.ocarto.2026.100785

**Published:** 2026-03-19

**Authors:** T.A. van Zadelhoff, E.H.G. Oei, W.E. van Spil, S.M.A. Bierma-Zeinstra, S.F. Madsen, A.C. Bay-Jensen, M. van Middelkoop, R.A. van der Heijden

**Affiliations:** a Department of Radiology and Nuclear Medicine, Erasmus University Medical Center Rotterdam, Rotterdam, the Netherlands; b Department of Rheumatology, Dijklander Hospital, Hoorn, the Netherlands; c Department of Rheumatology & Clinical Immunology, University Medical Center Utrecht, Utrecht, the Netherlands; d Department of General Practice, Erasmus MC Medical University Center Rotterdam, Rotterdam, the Netherlands; e Nordic Bioscience, Herlev Hovedgade 207, DK-2730 Herlev, Denmark; f Department of Biomedical Sciences, University of Copenhagen, Copenhagen, Denmark; g Department of Radiology, University of Wisconsin-Madison, Madison, WI, USA

**Keywords:** Patellofemoral pain, Serological biomarkers, Imaging dynamic contrast enhanced mri

## Abstract

**Purpose:**

The inflammatory, angiogenic and neurogenic pathways of osteoarthritis (OA) might already be present in patients with patellofemoral pain (PFP), a supposed precursor of OA. In this study, differences in serum levels of biomarkers of inflammation, angiogenesis and neurogenesis were compared between PFP patients and healthy controls. Furthermore, we aimed to determine the associations between serum marker levels and both clinical and MRI based inflammatory features.

**Methods:**

In a post hoc analysis of data from a prospective case-control study, a wide range of serum levels of markers of inflammation, angiogenesis and neurogenesis were measured in 44 PFP patients and 50 healthy controls aged 18–40 years. Clinical measures included the NRS for knee pain intensity, duration of complaints and pressure pain thresholds. Morphologic and dynamic contrast enhanced MRI of the knee were performed to assess inflammatory features and quantitative perfusion parameters. Differences in biomarker levels between patients and controls were assessed and additional analyses in the patient group were performed to study associations between serum biomarkers and inflammatory MR features and clinical parameters.

**Results:**

There were no significant differences between patients and controls in serum biomarker levels. Additional analyses in the patient group revealed no associations between serum markers and inflammatory MR features and clinical parameters.

**Conclusion:**

Our results were unable to confirm the hypothesis that inflammation, angiogenesis or neurogenesis plays a role in PFP pathophysiology.

## 1 Introduction

Patellofemoral pain (PFP) is a common knee condition among adolescents and young adults, characterized by retro- and peripatellar pain. The exact pathobiology of PFP remains unknown, but it is hypothesized to be a precursor of knee osteoarthritis (OA) [1,2].

There has been an increased interest in the potential of biochemical markers to predict, diagnose and monitor OA [[3], [4], [5]]. These biomarkers are intended to be used for tracking various processes in the pathophysiology of OA like inflammation, cartilage degeneration, angiogenesis, and neurogenesis [3]. Several serum markers are already increased in early-stage OA when no or minimal structural changes to the knee joint are visible on radiographs [6]. C-reactive protein metabolite (CRPM), a biomarker for inflammation, is associated with OA progression and people with higher CRPM levels are more likely to develop new-onset OA [7]. Therefore, it has been suggested that CPRM could be a marker for early identification of OA patients [7]. Vascular endothelial growth factor (VEGF), a mediator that potentially could be used as a biomarker that promotes angiogenesis, is correlated with the severity of OA [8]. Besides inflammatory and angiogenic markers, nerve growth factor (NGF) is also increased in the subchondral bone of OA patients and consequently it has been identified a specific target for OA treatment [9,10]. Since PFP is considered a precursor to OA, hypothetically, inflammatory, angiogenic and neurogenic biomarker alterations are already present in PFP, like they are in early OA. However, biomarker research in PFP is less abundant except for one study showing that NGF was increased in PFP patients with more pain due to patellofemoral malalignment [11].

The infrapatellar fat pad (IPFP), also known as Hoffa's fat pad, has been identified as a possible source of pain in both PFP and OA [12,13]. This fat pad is a richly innervated and highly vascularized structure anterior in the knee joint. Besides its biomechanical role it is thought to be a reservoir of immune cells responsible for the production of inflammatory cytokines, such as tumor necrosis factor-alfa (TNFα) and interleukines [14]. This IPFP-mediated inflammatory pathway has been implicated in the pathophysiology of OA and may similarly drive peripheral sensitization and symptom severity in PFP, suggesting that greater IPFP synovitis could be associated with increased sensitization and decreased function [[14], [15], [16], [17], [18]]. MRI enables assessment of IPFP features supposedly linked to inflammation, such as effusion synovitis or IPFP edema, also known as IPFP synovitis or Hoffa synovitis [19]. Dynamic contrast enhanced (DCE) MRI visualizes increased perfusion of the IPFP, as surrogate marker of inflammation [20]. This enables direct investigation of the potential relationship between imaging-defined IPFP synovitis and biochemical changes in PFP patients. This could provide an imaging biomarker for disease progression, since classic imaging features, like cartilage loss, are not yet present on x-ray and MRI [21,22].

Biochemical markers reflecting inflammatory, angiogenic and neurogenic processes may also capture underlying pathophysiological processes that contribute to muscle inhibition, altered motor control and reduced load tolerance, thereby influencing functional performance. Exploring the relationship between these biochemical markers and knee function in PFP could clarify whether worse function is associated with a more pronounced pro-inflammatory profile. In addition, exploration of the relationship between these biochemical markers and the pressure pain threshold (PPT), a measure of altered pain processing known to be aberrant in PFP patients [23], may shed light on potential underlying pathophysiological processes, such as low-grade inflammation, altered neuroimmune signaling and vascular changes relevant to nociceptor sensitization.

Identification of inflammation at the early stage of the PFP-OA continuum could be crucial for early treatment. However, it is unknown whether biochemical changes are already present in PFP patients. Therefore, the aim of this study was to determine whether there is a difference in biomarker levels for inflammation, angiogenesis and neurogenesis between PFP patients and healthy controls. We hypothesized that biomarkers reflecting inflammation, angiogenesis and neurogenesis are already elevated in PFP patients compared to healthy controls. An additional aim was to explore whether an association exists between inflammatory MRI features, clinical symptoms and serum markers for inflammation, angiogenesis and neurogenesis in the patient group.

## 2 Methods

For the current study, we conducted a post hoc analysis using data of adult participants of a prospective case-control study [23]. Patients in the original study were included between January 2013 and September 2014. They were eligible if they were 14–40 years of age, diagnosed with PFP by their general practitioner, physiotherapist or sports physician based on the presence of at least three of the following symptoms: retro- or peripatellar pain while walking up or down stairs, squatting, running, cycling, and sitting with knees flexed for a prolonged period of time, and grinding of the patella. A minimum symptom duration of 2 months and a maximum of 2 years was used. Patients were excluded if they had other defined pathological conditions of the affected knee such as patellar tendinopathy or osteoarthritis, if the onset of PFP occurred after trauma, or if they had previous knee injuries or surgery or previous episodes of PFP that occurred more than 2 years ago. Exclusion criteria for healthy controls were: knee pain, injury or surgery of any knee, a self-reported history of PFP and symptoms of patellar tendinopathy. Blood samples and DCE-MRI data were obtained only for the adults. The study was approved by the Medical Ethical Committee of Erasmus MC. All patients willing to participate signed an informed consent form (Medical Ethical Committee protocol no. MEC-2012-342).

### 2.1 Clinical symptom assessment

Clinical symptom assessment was performed at baseline. Patients were asked to fill in the anterior knee pain scale [24]. This questionnaire consists of 13 questions related to anterior knee pain with a total score of 0–100, with higher scores reflecting more subjective symptoms and functional impairment. Other clinical symptoms documented were knee pain intensity during rest and during exercise (numeric rating scale, range 0–10), duration of symptoms and bilateral complaints (yes/no). To measure the presence of altered pain processing, PPTs of the affected knee and contralateral forearm were measured using a handheld dynamometer (Biometrics MicroFET 2, Almere, The Netherlands). The measurement method has been described previously and was demonstrated to be reliable [25,26]. Measurements were collected in newton per square centimer (N/cm^2^).

### 2.2 Serum marker analysis

Data was collected between January 2013 and September 2014. At baseline, serum samples were taken from all participants simultaneous to the MRI acquisition. The serum separator tubes were centrifuged at 2000 g for 10 min. The resulting serum was aliquoted and immediately stored at −80 °C until use. All biomarkers used in this study are shown in Table 1. CRPM and VICM measurements were done using a validated ELISA (Nordic Bioscience, Herlev, Denmark) [27,28]. CXCL-10, VEGF, TNF-α and IL1-β were measured using Quantikine ELISA according to the manufacturer's analysis protocol (R&D Systems, Minneapolis, MN, USA). NGF was measured using ELISA according to the manufacturer's analysis protocol (Sigma Aldrich, Saint Louis, Missouri, USA). For VICM, CRPM and VEGF, all patients had biomarker levels between the lower and upper limit of measurement range (LLMR and ULMR). For CXCL-10, NGF, IL1-β and Tumor necrosis factor-α (TNF-α) there were 1, 44, 87 and 68 subjects, respectively, with an outcome at the LLMR or ULMR. NGF showed a large proportion of measurements outside the measurement range (LLMR-ULMR), but also more than half within the measurement range. In an effort to determine the true concentration of these samples, a two-fold dilution was performed which resulted in two additional measurements between the LLMR and ULMR. Since the measurements under the LLMR or above the ULMR could still be clinically relevant we decided to perform two analyses for NGF: one with all detectable samples and an additional one in which samples at the LLMR were assigned a score of 20% below the LLMR and the samples at the ULMR were assigned a score of 20% above the ULMR. In IL1-β and TNF-α almost all measurements were below the LLMR and were therefore not used for further statistical analysis.

**Table 1:** All serum markers analyzed.

Serum biomarker	Description	Biological process
CRPM	Fragment of CRP derived by MMP. Assumed to be indicative of more chronic inflammation opposed to acute inflammation than CRP.	Inflammation
CXCL-10/IP-10	The human interferon-inducible protein 10 (IP-10 or CXCL-10) is a chemokine of the CXC family. A unique feature of members of this chemokine family is that they have pro-inflammatory properties and act as modulators of angiogenesis in conditions such as wound healing, ischemia and neoplasia.	Inflammation and angiogenesis
Vascular endothelial growth factor (VEGF)	Endothelial cell-specific angiogenic factor. VEGF is expressed in articular cartilage during growth plate development and endochondral ossification	Angiogenesis
Citrullinated and MMP-degraded vimentin (VICM)	Released by activated macrophages and therefore a biomarker for macrophage activity.	Inflammation
Nerve growth factor (NGF)	Involved in the new growth of nerve cells and their maintenance. Overexpressed in many inflammatory and degenerative rheumatic disease.	Neurogenesis
Interleukin-1β (IL-1β)	Major inflammatory mediator. Assumed to contribute to pain [38].	Inflammation
Tumor necrosis factor-α (TNF-α)	Major inflammatory mediator. Able to induce inflammation, fever and apoptotic cell death.	Inflammation

### 2.3 MRI acquisition

MRI was performed with a 3 T MRI system (Discovery MR750, GE Healthcare, Milwaukee, WI) and a dedicated 8-channel knee coil (InVivo, Gainesville, FL). The DCE MRI sequence was a 3D spoiled gradient-echo acquired in the sagittal plane, consisting of 35 phases of 10 s. Intravenous gadolinium contrast administration (0.2 mmol/kg Magnevist; Bayer; Berlin; Germany) with a 2 ml/s injection speed started after the first phase. MRI parameters were: in-plane pixel resolution 1.5 mm, slice thickness 5 mm, field of view 380 × 380 × 70 mm, acquisition matrix 256 × 128, 14 sagittal slices, 70% sampling in the phase direction, TE = 1.7 ms, TR = 9.3 ms, FA = 30°.

### 2.4 (Semi)Quantitative MRI analysis

Semiquantitative analysis of effusion and Hoffa synovitis was done using the MRI Osteoarthritis Knee Score, a scoring tool with excellent reliability [19]. According to the MOAKS, Hoffa synovitis is defined as a diffuse hyperintense signal in the IPFP assessed on fat suppressed T2 or PD weighted sequences. Effusion synovitis is defined as hyperintense fluid signal in and around the patellofemoral articular cavity on an axial fat suppressed T2 weighted sequence. Effusion and Hoffa synovitis were rated on a 0–3 scale.

The perfusion parameters were calculated as previously described [29]. In short, the volume of interest was the entire IPFP. The Horos software package (Horosproject.org, USA) was used to delineate the volume of interest. The DCE add-on tool for Horos provided the perfusion parameters. The extended Tofts' model was used to fit the pharmacokinetic model. The following extended Tofts’ parameters were included in this study: *K*^trans^, which represents the transfer constant from the vascular compartment to the tissue compartiment, and V_p,_ which is the vascular fraction [30]. In case of inflammation, a higher perfusion is anticipated, reflected by more blood flowing into the tissue (*K*^trans^) and/or more or dilated blood vessels (V_p_). In case of angiogenesis/neurogenesis, blood vessel formation occurs, which might be reflected by an increase in V_p_.

### 2.5 Statistical analysis

This is a post hoc analysis of serum biomarkers in participants recruited in a prospective case-control study. Normality of all variables was tested using the Shapiro-Wilk test. Differences between groups were tested using the χ^2^ test for categorical variables. For continuous variables the independent samples *t*-test was used if normally distributed. If non-normally distributed the Mann-Whitney *U* test was used. ANCOVA was used to compare serum markers between patients and controls. Analyses were adjusted for age, sex and BMI. If non normally distributed, variables were log-transformed to allow ANCOVA and regression analysis. The mean and confidence intervals were back-transformed to the original scale for interpretability, while the regression coefficients (betas) were not, as they represent changes on the transformed scale. Back-transforming the betas would distort the interpretation of the variable relationships in the model.

Additional analyses were performed within the PFP patient group. Multiple linear regression analyses were used to determine the association between serum markers and the DCE-MRI parameters, AKPS, NRS and PPTs, respectively. All were adjusted for age, sex and BMI. Due to multiple testing Bonferroni adjustment was applied wherein the p-value cutoff was set to 0.05 divided by the number of tests in that particular analysis. All analyses were performed using SPSS (version 25, IBM, USA).

## 3 Results

### 3.1 Study sample

134 subjects aged 14 to 40 were recruited for the original study. Blood samples and DCE-MRI data were obtained only for the adults. This adult group consisted of 94 individuals, of which 44 were PFP patients and 50 controls. For 9 patients DCE-MRI data was not available due to errors during acquisition. Due to a human error, one box with 24 samples (6 patients and 18 controls) was lost in 2020 when the measurements were done. This box was retrieved in 2023 from a freezer. Identical batch numbers were still available for the VICM, CRPM, VEGF, CXCL-10 and IL1-beta assays that were used to analyze the retrieved samples and all analyses were performed according to protocol. Additional incurred sample reanalysis using in-study bridging samples were performed for CRPM and VEGF with a correction factor of 1.36 and 1.23, respectively. For NGF and TNF-α additional analysis of the 24 retrieved samples was not possible since the same batch numbers for the analysis kits were unavailable.

There was no significant difference in age (p = 0.68) and sex (p = 0.44) between patients and controls. BMI at baseline was significantly different between groups (Patient: 24.86; control: 23.23; mean difference 1.63 kg/m^2^, p = 0.02). Baseline characteristics can be found in Table 2.

**Table 2:** Baseline characteristics.

		Patient (n = 44)	Control (n = 50)	P-value
Female sex, n (%)		21 (47.70)	26 (52.00)	0.68
Age, mean (SD)		26.76 (5.77)	25.94 (4.42)	0.44
BMI (kg/m^2^), mean (SD)		24.86 (3.67)	23.23 (2.83)	0.02
Duration of complaints (months), mean (SD)		11.01 (6.44)	NA	NA
Pain (NRS) during rest, mean (SD)		4.00 (2.61)	NA	NA
Pain (NRS) during strain, mean (SD)		6.32 (2.31)	NA	NA
AKPS score, mean (SD)		68.84 (11.30)	NA	NA
Bilateral complaints, n (%)		19 (43.2)	NA	NA
Effusion (medium to large), n (%)	Physical amount	7 (16%)	3 (6%)	0.17
	Small	33 (75%)	38 (76%)	
	Medium	4 (9%)	9 (18%)	
Hoffa synovitis (moderate to severe), n (%)	Normal	19 (43%)	21 (42%)	0.98
	Mild	23 (52%)	27 (54%)	
	Moderate	2 (5%)	2 (4%)	
PPT affected knee (N/cm^2^), mean (SD)		42.25 (15.80)	52.60 (14.54)	0.001
PPT contralateral arm (N/cm^2^), mean (SD)		50.97 (13.41)	56.42 (14.25)	0.06

### 3.2 Serum markers

All biomarkers were non-normally distributed. For IL-1β 87 out of 94 (92.6%) and for TNF-α 68 out of 70 (97.1%) participants demonstrated serum concentrations which were below the LLMR similar to healthy controls according to the manufacturer. IL-1β and TNF-α were omitted from any further statistical analysis.

VICM was numerically higher in patients (11.7 ng/mL) compared to controls (8.6 ng/mL), however this difference was not statistically significant after Bonferroni correction. There were also no statistically significant differences between patient and controls for any other serum marker (Table 3).

**Table 3:** Serum markers (VICM and CRPM in ng/ml. VEGF, CXCL-10 and NGF in pg/ml) within the detection range in patients and controls. The ANCOVA was adjusted for age, sex and BMI. The data was log transformed to enable the analysis. Means and confidence intervals shown are back transformed. §: Not enough valid cases. Bonferroni corrected α = 0.007.

	Patient	Mean (95% CI)	Control	Mean (95% CI)	B (95% CI)	Partial ƞ^2^	P-value
	N		N				
VICM	44	11.67 (9.35–14.57)	50	8.84 (7.18–10.88)	0.28 (−0.03–0.59)	0.005	0.08
CRPM	44	9.60 (8.76–10.53)	50	9.60 (9.43–11.20)	−0.07 (−0.20–0.06)	0.026	0.30
VEGF	44	259.56 (208.30–323.44)	50	213.15 (173.47–261.91)	0.20 (−0.11–0.50)	0.01	0.20
CXCL-10	44	87.97 (74.96–103.23)	50	80.56 (69.41–93.50)	0.09 (−0.14–0.31)	0.005	0.44
IL-1β	6	1.61 (0.98–2.65)	1	3.09 (§)	(§)		(§)
TNF-α	1	33.12 (§)	1	15.96 (§)	(§)		(§)
NGF	13	457.6 (192.87–1085.72)	15	579.98 (259.82–1294.66)	−0.24 (−1.42–0.95)	0.007	0.68

### 3.3 Additional analyses in the patient group

All 44 PFP patients had complete morphological MRI data and clinical parameters and 35 had correctly acquired DCE-MRI data. There was no statistically significant or clinically relevant association between serum marker levels and quantitative DCE-MRI perfusion parameters (*K*^*trans*^, *V*_*p*_), or clinical features such as AKPS, duration of complaints, NRS or PPT (Table 4). The effect size (partial ƞ^2^) was low for all serum markers, ranging from 0.005 to 0.026.

**Table 4:** Multiple linear regression analysis with K^trans^, V_p_, AKPS, pain during rest, pain during strain, PPT of the contralateral arm or PPT of the affected knee as the dependent variable and the serum markers (VICM and CRPM in ng/ml. VEGF, CXCL-10 and NGF in pg/ml) as independent variables adjusted for age, sex and BMI. Bonferroni corrected α = 0.008.

Dependent variable: *K*^*trans*^	N	B	95% CI	P
VICM	35	−0.01	−0.02–0.01	0.32
CRPM	35	0.01	−0.03–0.04	0.60
VEGF	35	−0,0002	−0.001–0.001	0.57
CXCL-10	35	−0.002	−0.01–0.01	0.53
NGF including LLMR and ULMR	29	9.92E-6	0.00–0.00	0.50
NGF excluding LLMR and ULMR	9	3.33E-5	0.00–0.00	0.60

Dependent variable: *V*_*p*_		B		P

VICM	35	−0.003	−0.02–0.02	0.79
CRPM	35	0.001	−0.06–0.06	0.98
VEGF	35	0.0004	−0.001–0.001	0.37
CXCL-10	35	−0.003	−0.01–0.01	0.42
NGF including LLMR and ULMR	29	−8.89E-6	0.00–0.00	0.72
NGF excluding LLMR and ULMR	9	0.00	0.00–0.001	0.15

Dependent variable: AKPS		B		P

VICM	44	0.18	−0.13–0.48	0.24
CRPM	44	0.06	−0.89–0.99	0.89
VEGF	44	0.01	−0.01–0.02	0.37
CXCL-10	44	−0.01	−0.04–0.02	0.63
NGF including LLMR and ULMR	38	0.00	0.00–0.001	0.47
NGF excluding LLMR and ULMR	13	0.00	−0.004–0.004	0.83

Dependent variable: Duration of complaints		B		P

VICM	44	0.01	−0.18–0.20	0.86
CRPM	44	0.09	−0.47–0.65	0.75
VEGF	44	0.003	−0.01–0.01	0.47
CXCL-10	38	−0.01	−0.03–0.01	0.52
NGF including LLMR and ULMR	38	−4.26E-5	−0.001–0.00	0.85
NGF excluding LLMR and ULMR	13	5.07E-5	0.00–0.00	0.65

Dependent variable: Pain during rest		B		P

VICM	44	−0.04	−0.11–0.03	0.28
CRPM	44	0.12	−0.10–0.34	0.27
VEGF	44	−0.003	−0.01–0.001	0.15
CXCL-10	44	0.001	−0.01–0.01	0.91
NGF including LLMR and ULMR	38	−1.80E-5	0.00–0.00	0.80
NGF excluding LLMR and ULMR	13	0.00	−0.001–0.001	0.47

Dependent variable: Pain during strain		B		P

VICM	44	−0.04	−0.10–0.03	0.31
CRPM	44	−0.14	−0.34–0.06	0.17
VEGF	44	−0.0003	−0.004–0.003	0.86
CXCL-10	44	0.001	−0.01–0.01	0.74
NGF including LLMR and ULMR	38	7.16E-5	0.00–0.00	0.37
NGF excluding LLMR and ULMR	13	0.00	−0.001–0.00	0.65

Dependent variable: PPT contralateral arm		B		P

NGF including LLMR and ULMR	38	0.00	−0.001–0.001	0.46
NGF excluding LLMR and ULMR	13	−9.83E-5	−0.004 - 0.004	0.96

Dependent variable: PPT affected knee		B		P

NGF including LLMR and ULMR	38	0.00	−0.001–0.001	0.59
NGF excluding LLMR and ULMR	13	0.00	−0.003–0.003	0.87

The mean concentration of serum biomarker levels of patients and controls with different grades of synovitis on MRI are presented in Table 5. Patients with moderate Hoffa synovitis demonstrate higher levels of serum biomarker levels compared to patients with normal and mild Hoffa synovitis. Statistical tests were not performed due to the small number of patients with moderate Hoffa synovitis or medium effusion synovitis.

**Table 5:** Mean serum marker levels of patients and controls with and without morphological signs of inflammation on MRI. VICM and CRPM are given in ng/ml. VEGF, CXCL-10 and NGF are given in pg/ml.

Hoffa synovitis		Patient			Control	
	Normal (n = 19)	Mild (n = 23)	Moderate (n = 2)	Normal (n = 21)	Mild (n = 27)	Moderate (n = 2)
VICM	14.00	14.74	23.85	11.55	11.12	19.00
CRPM	9.76	10.26	15.40	10.48	10.79	8.63
VEGF	353.78	284.13	455.05	261.11	274.09	166.40
CXCL-10	92.42	112.01	221.53	82.31	97.16	135.75
NGF including LLMR + ULMR	3700.72 (n = 17)	1336.84 (n = 19)	6012.00 (n = 2)	1250.23 (n = 14)	1746.69 (n = 16)	1873.00 (n = 2)
NGF excluding LLMR + ULMR	1849.03 (n = 8)	222.39 (n = 5)	–	2169.90 (n = 7)	539.86 (n = 7)	3722.00 (n = 1)

		**Patient**			**Control**	
Effusion synovitis	Physical amount (n = 7)	Small (n = 33)	Medium (n = 4)	Physical amount (n = 3)	Small (n = 38)	Medium (n = 9)

VICM	20.52	13.88	12.72	28.78	10.76	9.52
CRPM	12.01	9.92	10.20	9.383	10.892	9.63
VEGF	309.90	321.72	345.29	331.91	283.23	162.00
CXCL-10	190.83	96.00	67.88	108.09	83.18	126.46
NGF including LLMR + ULMR	3347.90 (n = 5)	2863.00 (n = 29)	142.45 (n = 4)	24.00 (n = 1)	1556.52 (n = 23)	1671.53 (n = 8)
NGF excluding LLMR + ULMR	2345.74 (n = 2)	1071.50 (n = 10)	497.80 (n = 1)	–	1814.16 (n = 13)	623.10 (n = 2)

Patients with knee pain while sitting showed no significant difference in serum marker levels compared to patients without knee pain while sitting. The same was observed for patients with bilateral complaints versus those without (Table 6). Patients with bilateral complaints showed a notable higher VEGF concentration compared to patients without bilateral complaints, but this difference was not statistically significant.

**Table 6:** Difference in serum markers between patients with and without sitting pain and bilateral complaints. The ANCOVA was adjusted for age, sex and BMI. The data was log transformed to enable the analysis. Serum markers are given as mean (95% CI). Means and confidence intervals shown are back transformed. Bonferroni corrected α = 0.0125.

	Sitting pain (n = 8)	No sitting pain (n = 27)	B (95% CI)	P-value
VICM	12.3 (9.49–15.8)	11.82 (7.24–19.3)	0.04 (−0.53–0.6)	0.90
CRPM	9.87 (8.5–11.36)	8.33 (6.36–11.02)	0.16 (−0.16–0.48)	0.31
VEGF	265.07 (198.34–357.81)	314.19 (179.47–555.57)	−0.17 (−0.82–0.48)	0.60
CXCL-10	74.44 (65.37–84.77)	67.36 (52.46–85.63)	0.1 (−0.18–0.39)	0.47

	Bilateral complaints (n = 19)	No bilateral complaints (n = 25)	B (95% CI)	P-value

VICM	12.06 (8.76–16.78)	12.06 (9.12–15.8)	−0.01 (−0.46–0.45)	0.97
CRPM	9.49 (8–11.36)	9.87 (8.5–11.47)	0.04 (−0.2–0.29)	0.73
VEGF	340.36 (235.1–492.75)	208.51 (152.93–287.15)	−0.49 (−1.01–0.03)	0.06
CXCL-10	76.71 (58.56–100.48)	89.12 (70.11–112.17)	0.15 (−0.24–0.53)	0.44

## 4 Discussion

The primary aim of this study was to determine if PFP patients had altered inflammatory, angiogenic and neurogenic biomarker levels compared to healthy controls. We found no statistically significant differences in CRPM, CXCL-10, VEGF, VICM or NGF between PFP patients and healthy controls. IL-1β and TNF-α were both below the lowest measurable detection level similar to healthy controls according to the manufacturer. Besides no difference, effect sizes were low for all serum markers. Additional analyses in the patient group revealed no elevated serum marker levels in those with specific clinical signs or MRI features related to inflammation. These findings suggest that, at least in a relatively young PFP population with symptoms of up to two years, systemic inflammatory, angiogenic and neurogenic activity is not different from that of pain-free individuals. This challenges the hypothesis that PFP in this stage already exhibits the systemic biomarker profile typically observed in early knee OA and underscores the need to refine which PFP subgroups might show biologically detectable alterations.

In contrast to the abundance of biomarker research in OA, little research has yet been performed in serum marker levels in a PFP population. In a study by Witoński et al. TNF-α expression in the IPFP and synovium was increased in patients with anterior knee pain compared to healthy controls [31]. One important reason we did not find any significant difference between PFP patients and healthy controls might be that biomarkers were measured in the serum and not in knee joint tissues or fluids, potentially leading to measurements below the LLMR obscuring small differences and making the analyses more prone to distortion from other sources of biomarker release.

Recent research by Bolgla et al. demonstrated elevated MMP-9, a biomarker for inflammation, in young females with PFP when compared to healthy controls [32]. Although MMP-9 is a different inflammatory biomarker than those assessed in our study, these findings supported the hypothesis that inflammation contributes to PFP. In contrast, we did not observe elevated levels of CRPM, CXCL-10 or VICM in our PFP cohort compared to healthy controls, which suggests that systemic inflammatory changes may not be uniformly present across all PFP populations or may only be detectable for specific markers such as MMP-9. Future studies may therefore need to adopt broader biomarker panels and stratify PFP patients by sex, symptom duration and structural features to determine which inflammatory signatures, if any, characterize distinct PFP phenotypes.

Elevated serum markers have been observed in patients with OA. For instance, CRPM was increased in lower grade radiographic OA compared to higher grade [33]. Reports on the association between CRPM and pain intensity in OA patients vary, with reports of both no association and reports of a negative association with pain intensity [[33], [34], [35]]. In this study we found a negative but non-significant association between CRPM serum level and pain intensity during strain. Although this trend is consistent with reports of a negative association between CRPM and pain intensity in OA, the absence of statistical significance in our cohort preclude firm conclusions.

NGF plays a role in the development of sensory neurons and NGF levels are elevated in OA patients compared to healthy controls [36]. Inhibition of NGF reduces pain symptoms of patients with symptomatic OA [37]. In this study NGF was not elevated in PFP patients compared to controls. Furthermore, NGF serum concentration varied greatly in this population with patients both at the LLMR (32) and at the ULMR (10). Based on our data we were unable to confirm our hypothesis that the pain perceived in patients with PFP is explained by an increase in neural growth. The absence of an association between PPT and NGF levels supports this statement. This suggests that systemic NGF may not be a sensitive marker of nociceptive processes in young PFP patients, despite its established role in OA-related pain and the analgesic effects of NGF inhibition in that context [37,38].

In the original study, focusing solely on MR perfusion parameters and volume of the IPFP, patients with effusion synovitis did show a higher perfusion of the IPFP [29]. We expected that patients with increased MR perfusion parameters would show increased angiogenesis and inflammatory biomarkers, however we were unable to demonstrate this. With regard to VEGF, patients with bilateral complaints showed a higher VEGF concentration compared to patients without bilateral complaints, which might point to a systemic pathway, though the difference was not statistically significant. Unfortunately, in this study we were only able to evaluate associations in the patient group in an explorative way due to the small sample sizes. Therefore, no firm conclusions can be drawn yet. Nonetheless, these insights can guide future studies, which might be able to confirm certain patient subgroups.

To our knowledge this is the first study in PFP patients in which CRPM, CXCL-10, VEGF, VICM, NGF, IL-1β and TNF-α were determined and compared to healthy controls. Therefore, this was a unique opportunity to discover if serum biomarkers were elevated in this patient population. Furthermore, an extensive range of clinical tests and imaging, including advanced DCE-MRI, were acquired.

A limitation to this study is that part of the biomarker samples were analyzed at a later stage due to the loss of one sample box due to a human error. After the box was retrieved the analysis of the remaining samples could fortunately be performed with identical kits for most biomarkers leading to accurate results after minor corrections. However, analysis of the remaining samples for IL-1β and TNF-α was not possible, due to unavailability of identical kits. Nonetheless, almost all of the already analyzed samples were at the LLMR suggesting no elevated levels of these biomarkers in this population. A second potential limitation of this study could be the systemic measurement of biomarkers. With this approach there is always the chance that other processes not related to the pathology of interest influences the results. However, since our population was relatively young and healthy we do not suspect that other processes are prevalent. Furthermore, deriving synovial fluid is invasive and not preferred in the relatively young and healthy PFP population. Moreover, in OA, serum biomarkers have been shown to be elevated indicating the potential of serum biomarkers to capture joint related pathologies. A third potential limitation is the low prevalence of high-grade effusion and Hoffa synovitis in this population. Although patients with more severe Hoffa synovitis showed higher levels of serum biomarkers, statistical testing was not possible due to the small group size. Future research might focus on a slightly older patient group with a longer symptom duration, which are known to show more MR features [39]. Hypothetically, such a population might also have developed more severe tissue homeostasis imbalances, contrary to the current relatively young population with a maximum symptom duration of two years.

The menstrual cycle phase in female participants was not taken into account when collecting blood samples. For OA it is established that a decreasing estrogen level increase the risk for OA [40]. Furthermore, estrogen inhibits inflammation and chondrocyte degradation, delaying the onset of OA [41]. If PFP is a precursor to OA It is not unlikely that hormonal fluctuations during the menstrual cycle could have influenced the biomarker levels in our samples.

The average age of the PFP group was 26.76 and for the control group 25.94. This is a common mean age group in patellofemoral pain research, but limits the direct generalizability of our results to adults over 30 years, where pathophysiological mechanisms such as growth-related cartilage stress or degenerative changes start playing a role and could yield different biomarker patterns. Future research could focus on an older age group to see if a more pronounced biochemical marker pattern is present.

In conclusion, our results were unable to confirm the hypothesis that inflammation, angiogenesis or neurogenesis plays a role in PFP pathophysiology. Future research should elucidate whether this is due to PFP not having an inflammatory pathophysiology contrary to OA, or whether these imbalances only occur in those with a symptom duration longer than two years.

## Author contributions

TvZ and RvH were involved the analysis and primary draft of the manuscript. RvdH was involved in the data collection of all subjects. SM and ABJ performed the biomarker analyses. EO, MvM, SBZ, WEvS and RvdH had an essential role in the study design. All authors contributed to data interpretation and critical manuscript revision. All authors approved the final version.

## Role of the funding source

This research received no external funding.

## Conflict of interest

SBZ declares to have received consulting fees from Infirst HealthCare and Pfizer. ABJ declares to be full time employee and shareholder of Nordic Bioscience, Herlev Hovedgade 207, DK-2730 Herlev, Denmark. All other authors declare no competing interest.
